# Prostaglandin E_2_ sensitizes the cough reflex centrally via EP3 receptor-dependent activation of NaV 1.8 channels

**DOI:** 10.1186/s12931-021-01889-4

**Published:** 2021-11-18

**Authors:** Al-Shaimaa A. Al-Kandery, Muddanna S. Rao, Ahmed Z. El-Hashim

**Affiliations:** 1 Department of Pharmacology & Therapeutics, Faculty of Pharmacy, Kuwait University, Kuwait City, Kuwait; 2 Department of Anatomy, Faculty of Medicine, Kuwait University, Kuwait City, Kuwait

**Keywords:** PGE_2_, Cough, Central sensitization, EP1-4 receptors, TRPV1, TRPA1, TTX-sensitive channels and NaV 1.8 channels

## Abstract

**Background:**

Cough hypersensitivity is a major characteristic feature associated with several types of cough, including chronic cough, but its underlying mechanisms remain to be fully understood. Inflammatory mediators, such as prostaglandin E_2_ (PGE_2_), have been implicated in both peripheral induction and sensitization of the cough reflex. In this study, using a conscious guinea pig model of cough, we investigated whether PGE_2_ can sensitize the cough reflex via central actions and, if so, via which mechanisms.

**Methods:**

All drugs were administered by intracerebroventricular (i.c.v.) route and whole-body plethysmograph set-up was used for both induction, using aerosolized citric acid (0.2 M), and recording of cough. Immunohistochemistry was performed to confirm the expression of NaV 1.8 channels in the nucleus tractus solitarius (nTS).

**Results:**

We show that both PGE_2_ and the non-selective EP1/EP3 agonist, sulprostone, dose-dependently enhanced the citric acid-induced cough (P ≤ 0.001, P ≤ 0.01, respectively). Pretreatment with the EP1 antagonist, ONO-8130, did not affect the sulprostone-induced cough sensitization, whilst the EP3 antagonist, L-798,106, dose-dependently inhibited this effect (P ≤ 0.05). Furthermore, treatment with either the EP2 agonist, butaprost or the EP4 agonist, L-902,688, had no effect on cough sensitization. Additionally, pretreatment with either the TRPV1 antagonist, JNJ-17203212 or the TRPA1 antagonist, HC-030031, alone or in combination, nor with the NaV 1.1, 1.2, 1.3, 1.4, 1.6 and 1.7 channel blocker, tetrodotoxin, had any effect on the cough. In contrast, pretreatment with the NaV 1.8 antagonist, A-803467, dose-dependently inhibited this effect (P ≤ 0.05). Furthermore, NaV 1.8 channels were shown to be expressed in the nTS.

**Conclusion:**

Collectively, our findings show that PGE_2_ sensitizes the cough reflex centrally via EP3 receptor-dependent activation of NaV 1.8 but independently of TRPV1,TRPA1 and TTX-sensitive sodium channel activation. These results indicate that PGE_2_ plays an important role in central sensitization of the cough reflex and suggest that central EP3 receptors and/or NaVv 1.8 channels may represent novel antitussive molecular targets.

**Graphical Abstract:**

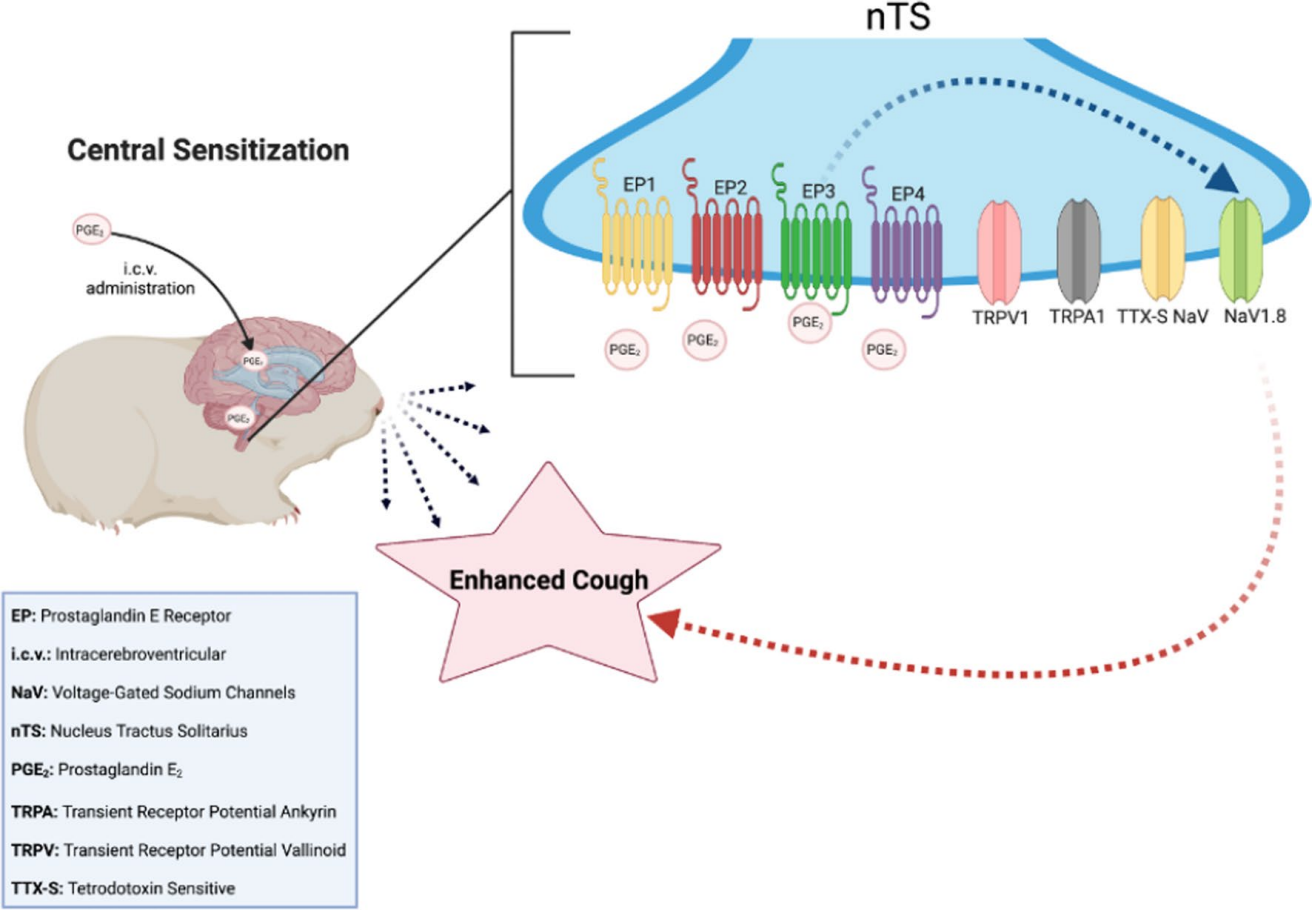

## Background

Cough, particularly that of a chronic nature, continues to be a challenging clinical condition with a high global prevalence leading to a significant economic expenditure and utilization of health care resources [1, 2]. Cough Hypersensitivity Syndrome (CHS) is a recently coined term to describe a chronic  exaggerated cough response, to numerous stimuli, which may exist as a distinct clinical entity [3]. The lack of effective cough therapy in the market, to date, reflects our incomplete understanding of cough mechanisms and related pathways that underlie CHS.

The role of the brainstem and higher brain regions in cough is particularly poorly understood, compared to that of the airways, possibly due to difficulty of access, complexity of the neuroanatomy and lack of appropriate animal models [4–9]. Notwithstanding, progress has been made recently in identifying some of the neuromediators, pathways and neuroplastic changes involved in cough, at both peripheral and central levels [10, 11].

There is a growing body of evidence suggesting that airway and central neuroplastic changes, driven by inflammation, are important drivers of cough. Interestingly, many of the inflammatory mediators involved in hyperalgesia, such as substance P (SP), nerve growth factor (NGF) and bradykinin (BK) also induce and/or sensitize the cough reflex [12–17] partly via activation and/or upregulation of ion channels. In particular, transient resistant potential (TRP) channels such as TRPV1 and TRPA1 have been reported to play key roles in sensitizing the cough reflex both peripherally and centrally [12, 13, 18, 19]. More recently, voltage-gated sodium channels (NaVs), mainly NaV 1.7, 1.8 and 1.9, which are expressed in airway sensory neurons, have been reported to be involved in the regulation of peripheral cough [20–22].

Prostaglandin E_2_ (PGE_2_), a major mediator of pain, has been demonstrated, paradoxically, to have both proinflammatory and protective bronchodilator effects in the respiratory system [23], particularly when given by inhalation [24, 25]. Moreover, several preclinical and clinical studies have shown that aerosolization of PGE_2_ can both induce and sensitize the cough reflex to citric acid and/or capsaicin [24, 26, 27]. Indeed, treatment with NSAIDs can reduce virally-induced cough and cough associated with angiotensin converting enzyme (ACE) inhibitors suggesting an important role for PGE_2_ in cough induced via these modalities [28, 29].

The actions of PGE_2_ are mediated predominantly through E-prostanoid (EP) receptors, which are classified into four distinct subtypes: EP1, EP2, EP3 and EP4 [30, 31]. EP receptors are 7-transmembrane G-protein coupled receptors (GPCR) that differ in their G-protein coupling and signaling cascade. The EP3 receptors exist in multiple splice variants produced by alternative splicing of the C-terminal tail. Whilst all the EP receptors have been reported to have airway effects, good evidence suggests that the EP3 receptor is the main receptor involved in PGE_2_-sensitization and induction of cough at the airway level [24]. However, whether PGE_2_ can sensitize the cough reflex via a central action has not been previously addressed.

Based on our previous findings with inflammatory mediators and the evidence supporting the role of PGE_2_ in peripheral induction and sensitization of the cough reflex, we proposed that PGE_2_ also sensitizes the cough reflex centrally. In this study, and using a conscious guinea pig model of cough, we investigated [1] whether PGE_2_ administered centrally can sensitize the cough reflex and if so, [2] what are the downstream signaling mechanisms involved.

## Materials and methods

### Ethical considerations

All the experimental protocols were approved by the Animal Ethics Committee in the Health Sciences Center at Kuwait University. In addition, all the experiments were conducted in accordance with international and Kuwait University guidelines and complied with the EU Directive 2010/63/EU for animal experiments and the National Institutes of Health Guide for the care and use of laboratory animals (NIH Publications No. 8023, revised 1978).

### Animals

Conscious, unrestrained adult Dunkin-Hartley guinea pigs, both males and females, weighing 400–600 g were used for all the experiments in this study. The in- house bred animals were generously supplied by the Animal Resources Center of Faculty of Medicine at Kuwait University and were housed in a fully controlled room in the animal facility maintained at 21–25 °C, relative humidity of 50% and 12-h light/dark cycle. Following cannulae implantation, all animals were placed individually in plastic cages on sawdust bedding to avoid removal or damage to the implanted cannula. The animals had unrestricted access to standard diet and tap water supplemented with multivitamins.

### Surgical implantation of chronic intracerebroventricular cannula

The surgical implantation procedures were performed as previously described [14]. Guinea pigs were first anesthetized with ketamine hydrochloride 80 mg/kg (Laboratories Sterop, Brussels, Belgium) and xylazine hydrochloride 6 mg/kg (Sigma-Aldrich, St Louis, MO, USA) administered intramuscularly (i.m.). Fifteen minutes thereafter, each animal was injected with the antibiotic enrofloxacin 0.25 mg/kg subcutaneously (s.c.) (Bayer AG, Berlin, Germany) and the pain control medication, tramadol hydrochloride 1 mg/kg (i.m.) (Sigma-Aldrich, St Louis, MO, USA). The fur over the guinea pig head was shaved off and the animal was then placed in a stereotaxic apparatus (David Kopf Instruments, Tujunga, CA, USA). The head was cleaned with betadine, a midline incision in the skin above the skull (2 cm) was made with a sharp surgical blade No. 20 (Feather Safety Razor, Osaka, Japan), and the skull was cleaned with 3% hydrogen peroxide (Sigma-Aldrich, St Louis, MO, USA) to ensure that all connective tissues were removed from the surface of the skull. A 20-gauge stainless steel guide cannula and its dummy cannula, HTX-20 T and HTX-25R (Plastic one, Roanoke, VA, USA) were placed in the lowering arm of the stereotaxic apparatus. The cannula was moved in the following three dimensions relative to the bregma and corresponded to the left lateral ventricle: 2.0 mm anteroposterior (AP), 1.8 mm mediolateral (ML) and 4.8 mm dorsoventral (DV), which is based on previously published reports [14, 32, 33]. A small hole was then drilled in the skull, based on the determined coordinates. Two additional holes were drilled for two anchor screws (Stoelting, IL, USA). Dental cement (Stoelting, IL, USA) was used to fix the cannula in the predetermined coordinates. The animals were also treated with intramuscular tramadol hydrochloride (1 mg/kg) and subcutaneous enrofloxacin (0.25 mg/kg) for two consecutive days to relieve pain and prevent post-operative infections.

### Preparation of drugs and buffers

PGE_2_ (Cayman Chemical Company) was initially prepared by dissolving the drug in absolute ethanol and dilutions were made in artificial cerebrospinal fluid (ACSF) to yield the desired final concentrations. Sulprostone, butaprost, L-902,688, ONO-8130, L-798,106, A-803467 (Cayman Chemical Company), JNJ-17203212 (Tocris Bioscience) and HC-030031 (Sigma Aldrich) were initially dissolved in dimethyl sulfoxide (DMSO) and subsequent dilutions were made in ACSF to yield the desired final concentrations as shown in Table 1. The solubility of these different drugs in DMSO were different and this resulted in different percentage concentration of DMSO being used. However, it is important to note that, in each set of experiments, appropriate controls containing the right concentration of DMSO were used to allow proper scientific assessment of drug effects. Tetrodotoxin (Tocris Bioscience) was initially dissolved in ACSF to make 1 mM solution and subsequent dilutions were made in ACSF to yield the desired final concentration. Citric acid (Sigma Aldrich) was dissolved in phosphate buffer saline (PBS, Sigma Aldrich) to make 0.2 M solution. All drugs were freshly prepared for each experiment.

**Table 1:** Summary of the different experiments done in this study including the objectives of the experiments, treatments (with concentrations) and controls (with vehicles used to dissolve the agonists and antagonists) and the number of animals used in each group

Treatment/ Pretreatment	Objective	Control	Treatment Groups		
		Vehicle	Concentration 1	Concentration 2	Concentration 3
PGE_2_	To study the effect of treatment on citric acid-induced cough	10% ethanol in ACSF (n = 12)	0.3 mg/ml (n = 8)	0.6 mg/ml (n = 12)	1 mg/ml (n = 13)
Sulprostone Non-selective EP1/EP3 agonist	To study the effect of treatment on citric acid-induced cough	10% DMSO in ACSF (n = 9)	0.1 mg/ml (n = 8)	0.3 mg/ml (n = 7)	1 mg/ml (n = 9)
ONO-8130 EP1 receptor antagonist	To study the effect of pretreatment on sulprostone-enhanced citric acid-induced cough	70% DMSO in ACSF + sulprostone (1 mg/ml) (n = 10)	1 mg/mL + sulprostone (1 mg/ml) (n = 7)	5 mg/mL + sulprostone (1 mg/ml) (n = 6)	–
L-798,106 EP3 receptor antagonist	To study the effect of pretreatment on sulprostone-enhanced citric acid-induced cough	70% DMSO in ACSF + sulprostone (1 mg/ml) (n = 10)	2.5 mg/mL + sulprostone (1 mg/ml) (n = 7)	5 mg/mL + sulprostone (1 mg/ml) (n = 10)	–
Butaprost EP2 receptor agonist	To study the effect of treatment on citric acid-induced cough	10% DMSO in ACSF (n = 11)	0.3 mg/ml (n = 8)	1 mg/ml (n = 10)	–
L-902,688 EP4 receptor agonist	To study the effect of treatment on citric acid-induced cough	10% DMSO in ACSF (n = 7)	0.3 mg/ml (n = 9)	1 mg/ml (n = 8)	–
JNJ-17203212 TRPV1 channel antagonist	To study the effect of pretreatment on PGE_2_-enhanced citric acid-induced cough	70% DMSO in ACSF + PGE_2_ (1 mg/ml) (n = 10)	0.4 mg/mL + PGE_2_ (1 mg/ml) (n = 10)	1.3 mg/mL + PGE_2_ (1 mg/ml) (n = 9)	–
HC-030031 TRPA1 channel antagonist	To study the effect of pretreatment on PGE_2_-enhanced citric acid-induced cough	5% DMSO in ACSF + PGE_2_ (1 mg/ml) (n = 8)	0.02 mg/mL + PGE_2_ (1 mg/ml) (n = 7)	0.05 mg/ml + PGE_2_ (1 mg/ml) (n = 6)	–
Combination JNJ-17203212 + HC-030031	To study the effect of pretreatment on PGE_2_-enhanced citric acid-induced cough	70% DMSO in ACSF + PGE_2_ (1 mg/ml) (n = 7)	0.4 + 0.02 mg/mL + PGE_2_ (1 mg/ml) (n = 5)	1.3 + 0.05 mg/mL + PGE_2_ (1 mg/ml) (n = 6)	–
Tetrodotoxin (TTX) Tetrodotoxin-sensitive sodium channel antagonist	To study the effect of pretreatment on PGE_2_-enhanced citric acid-induced cough	99% ACSF + PGE_2_ (1 mg/ml) (n = 7)	0.015 µg/mL + PGE_2_ (1 mg/ml) (n = 7)	0.1 µg/mL + PGE_2_ (1 mg/ml) (n = 5)	–
A-803467 NaV 1.8 channel antagonist	To study the effect of pretreatment on PGE_2_-enhanced citric acid-induced cough	70% DMSO in ACSF + PGE_2_ (1 mg/ml) (n = 6)	5 mg/mL + PGE_2_ (1 mg/ml) (n = 5)	10 mg/mL + PGE_2_ (1 mg/ml) (n = 5)	–

### Intracerebroventricular drug administration

The drugs were administered to conscious and unrestrained guinea pigs on day 8 after cannula implantation as previously described [14, 32, 33]. Briefly, prior to each cough assessment experiment, the dummy cannula was removed from the guide cannula. The infusion cannula was then connected to the guide cannula and a vehicle/drug filled syringe fixed to a Harvard 33 Twin Syringe Pump (Harvard Apparatus, USA) via a polyethylene tubing (PE-60) (Small parts, INC, USA). Drugs were infused at a rate of 30 μl/h, with a maximum volume of 15 μl administered over a period of 30 min followed by a 15-min absorption period to prevent the backflow of the drug and allow the drug to equilibrate. In the experiments involving agonists and antagonists treatments, the agonist drug was administered 45 min after the antagonist pretreatment (30-min antagonist infusion followed by 15-min absorption phase). The accuracy of the cannula implantation was checked via the infusion of methylene blue at the end of some of the experiments and an 80% accurate implantation was noted.

### Citric acid challenge and measurement of cough

**Fig. 1:**
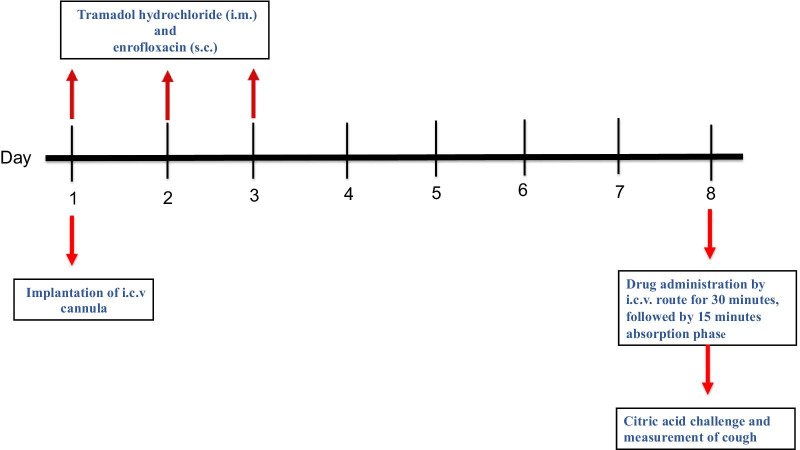
A schematic diagram for the overall timeline for the experimental protocol used to investigate PGE_2_-induced sensitization of the cough reflex. Intracerebroventricular (i.c.v.) cannulae were implanted and guinea pigs were then injected with tramadol (i.m*.*) and enrofloxacin (s.c.) for three consecutive days. After a one-week recovery period, drugs were infused through i.c.v. route for 30 minutes followed by a 15-minute absorption phase. In case of two drug treatments, the antagonist was infused first for 30 minutes followed by a 15-minute absorption phase, then the agonist was infused for 30 minutes followed by a 15 minute absorption phase. Animals were then challenged with 0.2 M citric acid for 10 minutes followed by a 10-minute post-nebulization recording period. Cough number was assessed over the entire 20-minute nebulization/recording period

The citric acid challenge and the assessment of cough were conducted using the Buxco whole body plethysmography (WBP) system (Buxco, Troy, NY, USA) as previously described [14, 34]. Fifteen minutes following the infusion of the drug, conscious and unrestrained guinea pigs were placed individually in the transparent plethysmography chambers. The animals were then exposed to an aerosolized 0.2 M aqueous citric acid (Sigma-Aldrich, St Louis, MO, USA), which is generated by the aerogen nebulizer. The bias flow generator, which is connected to the plethysmography chamber, was set to supply air at a rate of 3 L/min and withdraw air at a rate of 4 L/min. The number of coughs were recorded over a 20-min period, which was divided into 10-min of citric acid challenge followed by 10-min post-nebulization recording period, using the Buxco cough analyzer. The Buxco cough analyzer differentiates coughs from sneezes and has been reported to demonstrate > 99% correlation with manual cough counting [34]. The criteria and number of coughs were also confirmed by observing the characteristic opening of the mouth, forward movement coupled to the high sound produced by the guinea pigs as well as a defined pattern in the sound signal. The overall timeline of the experimental protocols used to investigate PGE_2_-induced sensitization of the cough reflex is shown in Fig. 1.

### Animal perfusion and qualitative immunohistochemistry (IHC) study for the expression of NaV 1.8 channels in the brainstem nucleus, nTS

Due to the scarcity of data for central expression of NaV 1.8 channels, a qualitative IHC studies were conducted to detect the expression of NaV 1.8 channels in the brainstem nucleus, nTS, in a few guinea pigs. The guinea pigs were anesthetized with ketamine hydrochloride 80 mg/kg (Laboratories Sterop, Brussels, Belgium) and xylazine hydrochloride 6 mg/kg (Sigma-Aldrich, St Louis, MO, USA) administered intramuscularly (i.m.) and perfused transcardially with freshly prepared 4% paraformaldehyde. Briefly, the heart was exposed and perfused with 150 mL of heparinized saline followed by 800 mL of 4% paraformaldehyde (in 0.1 M Phosphate buffer, pH 7.4). The brain was dissected out and post fixed for 48 h in the same fixative. Five mm length of medulla oblongata tissue starting from obex towards the spinal cord was processed for paraffin section cutting. Five-micron thick serial sections were cut and mounted on Poly-L-lysine coated glass slides. Serial brain sections were stained for cresyl violet staining and double immunofluorescence staining for NaV 1.8 and NeuN (marker for Neuron). Selected sections were incubated in a cocktail of polyclonal rabbit anti-NaV1.8 (1:250, NBP2-75,584, Novus Biologicals) and monoclonal mouse anti-NeuN-1 (1:500, ab104224, Abcam) antibodies overnight. The sections were then washed in PBS and treated with a cocktail of goat anti-rabbit DyLight-594 and horse anti-mouse DyLight 488 secondary antibodies (1:200, DI-2488–1.5, Vector Laboratories). Sections were washed and mounded with vectashield mounting media. Sections were observed and photographed in the confocal microscope.

### Animal euthanization

At the end of each experiment, animals were sacrificed by carbon dioxide (CO_2_) inhalation. The CO_2_ gas was delivered using a compressed gas cylinder, pressure regulator and flowmeter. The flow rate of CO_2_ was adjusted to 5 L/min and maintained until breathing, all muscle activity and signs of life are completely absent. This was followed by cervical dislocation to ensure death.

### Experimental protocols

The present study consisted of several experiments summarized in Table 1. In all experiments, animals were arbitrarily divided into control group and two or three treatment groups. Control groups were treated with 15 μl of different vehicles that were used to dissolve the agonists or antagonists. Animals in the treatment groups were treated with 15 μl of different concentrations of agonists and/or antagonists. It is noteworthy to point out that cough numbers are not affected, in either the agonist or antagonist experiments, by the different concentrations of the vehicle (DMSO; 5–70%).

### Statistical analysis

Statistical evaluation analysis was carried out with Microsoft Excel for Mac (Microsoft Software V 15.31, USA) and GraphPad Prism (GraphPad Software, San Diego, CA). The data were analyzed blindly and expressed as mean cough ± standard error of the mean (SEM) and represent the number of coughs during the 20-min period of assessment. All treatment groups were initially tested for normality using Shapiro–Wilk test of normality. Normally distributed data were analyzed by parametric one-way analysis of variance (ANOVA), followed by Dunnett's multiple comparisons test. Nonparametric data were analyzed using nonparametric Kruskal–Wallis ANOVA followed by Dunn’s multiple comparison. At P ≤ 0.05, differences were considered statistically significant. All the groups in each experiment were time-matched to ensure all treatment groups were exposed to the same experimental conditions.

## Results

### Effect of treatment with PGE_2_ on citric acid-induced cough

**Fig. 2:**
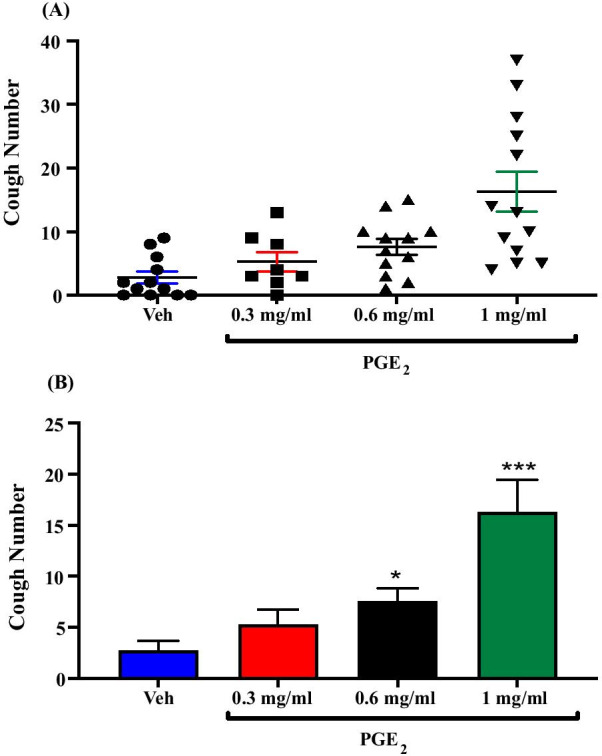
Effect of treatment with PGE_2_ on citric acid-induced cough. Treatment of guinea pigs with 15 μl of PGE_2_ (0.3 mg/ml; n = 8, 0.6 mg/ml; n = 12 and 1 mg/ml; n = 13, i.c.v.) resulted in a dose-dependent increase in citric acid-induced cough response compared to vehicle (n = 12). Data are plotted as a scatter graph **A** and a bar chart **B**, showing mean cough ± SEM. * and *** represent a statistically significant difference at P ≤ 0.05 and P ≤ 0.001, respectively, when compared to vehicle treated animals

Treatment of guinea pigs with PGE_2_; i.c.v. (0.3 mg/ml; n = 8, 0.6 mg/ml; n = 12 and 1 mg/ml; n = 13) resulted in a dose-dependent increase in citric acid-induced cough response compared to vehicle treated guinea pigs (n = 12). The mean cough ± SEM were the following: 5.25 ± 1.53 (0.3 mg/ml PGE_2_), 7.58 ± 1.28 (0.6 mg/ml PGE_2_), 16.31 ± 3.16 (1 mg/ml PGE_2_) and 2.75 ± 0.94 (vehicle). Both 0.6 mg/ml and 1 mg/ml resulted in a significant increase in citric acid-induced cough (> 175%, P = 0.035; > 490%, P = 0.0001, respectively). The data are presented in Fig. 2A, B. Since PGE_2_ at concentration of 1 mg/ml resulted in the highest enhancement of the citric acid-induced cough without any overt unwanted effects, this dose was used in the subsequent experiments in this study involving the use of PGE_2_.

### Effect of treatment with the non-selective EP1/EP3 agonist, sulprostone, on citric acid-induced cough

**Fig. 3:**
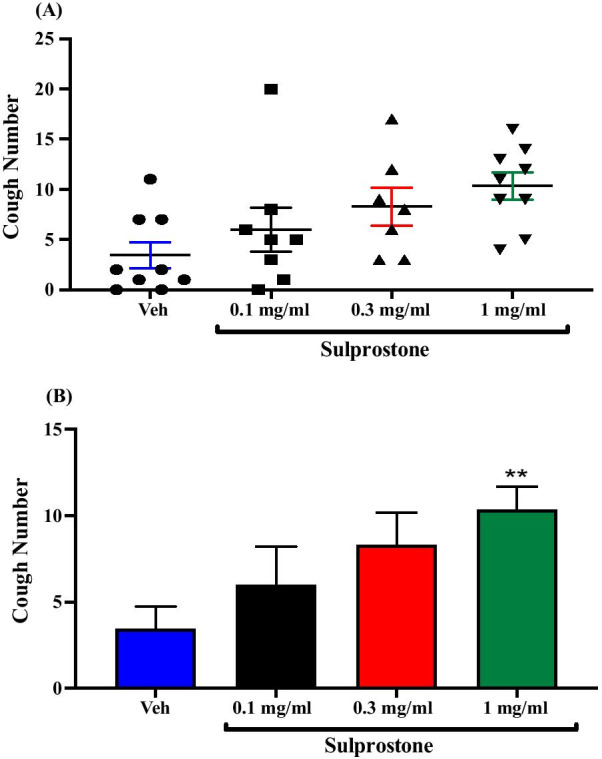
Effect of treatment with the non-selective EP1/EP3 agonist, sulprostone, on citric acid-induced cough. Treatment of guinea pigs with 15 μl of sulprostone (0.1 mg/ml; n = 8, 0.3 mg/ml; n = 7 and 1 mg/ml; n = 9, i.c.v.) resulted in a dose-dependent increase in citric acid-induced cough response compared to vehicle (n = 9). Data are plotted as a scatter graph **A** and a bar chart **B** showing mean cough ± SEM. ** represents a statistically significant difference at P ≤ 0.01 when compared to vehicle treated animals

Treatment of guinea pigs with the non-selective EP1/EP3 agonist, sulprostone; i.c.v. (0.1 mg/ml; n = 8, 0.3 mg/ml; n = 7 and 1 mg/ml; n = 9) resulted in a dose-dependent increase in citric acid-induced cough response compared to vehicle treated guinea pigs (n = 9). The mean cough ± SEM were the following: 6.00 ± 2.20 (0.1 mg/ml sulprostone), 8.29 ± 1.90 (0.3 mg/ml sulprostone), 10.33 ± 1.33 (1 mg/ml sulprostone) and 3.44 ± 1.30 (vehicle). Sulprostone at 1 mg/ml caused a significant increase in citric acid-induced cough (> 200%, P = 0.007). The data are presented in Fig. 3A, B. Since sulprostone at concentration of 1 mg/ml resulted in significant enhancement of the citric acid-induced cough, this dose was used in the subsequent experiments involving the use of sulprostone.

### Effect of pretreatment with the EP1 receptor antagonist, ONO-8130, on sulprostone-enhanced citric acid-induced cough

**Fig. 4:**
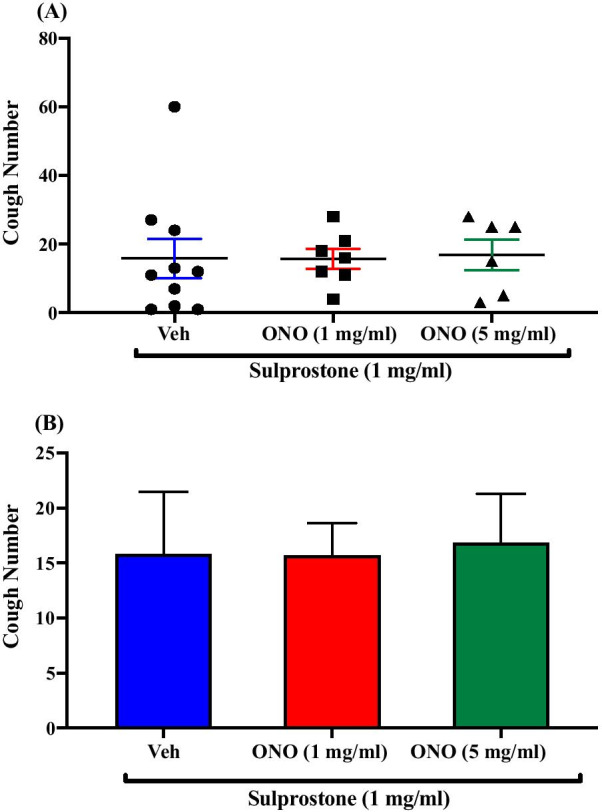
Effect of pretreatment with the EP1 antagonist, ONO-8130, on sulprostone-enhanced citric acid-induced cough. Pretreatment of guinea pigs with 15 μl of ONO-8130 (1 mg/ml; n = 7 and 5 mg/ml; n = 6, i.c.v.) did not affect the sulprostone-enhanced citric acid-induced cough response compared to vehicle (n = 10). Data are plotted as a scatter graph **A** and a bar chart **B** showing mean cough ± SEM

Pretreatment of guinea pigs with the EP1 antagonist, ONO-8130; i.c.v. (1 mg/ml; n = 7 and 5 mg/ml; n = 6) did not affect the sulprostone-enhancement of citric acid-induced cough response compared to vehicle pretreated guinea pigs (n = 10). The mean cough ± SEM were the following: 15.71 ± 2.92 (1 mg/ml ONO-8130), 16.83 ± 4.45 (5 mg/ml ONO-8130) and 15.80 ± 5.67 (vehicle). The data are presented in Fig. 4A, B.

### Effect of pretreatment with the EP3 receptor antagonist, L-798,106, on sulprostone-enhanced citric acid-induced cough

**Fig. 5:**
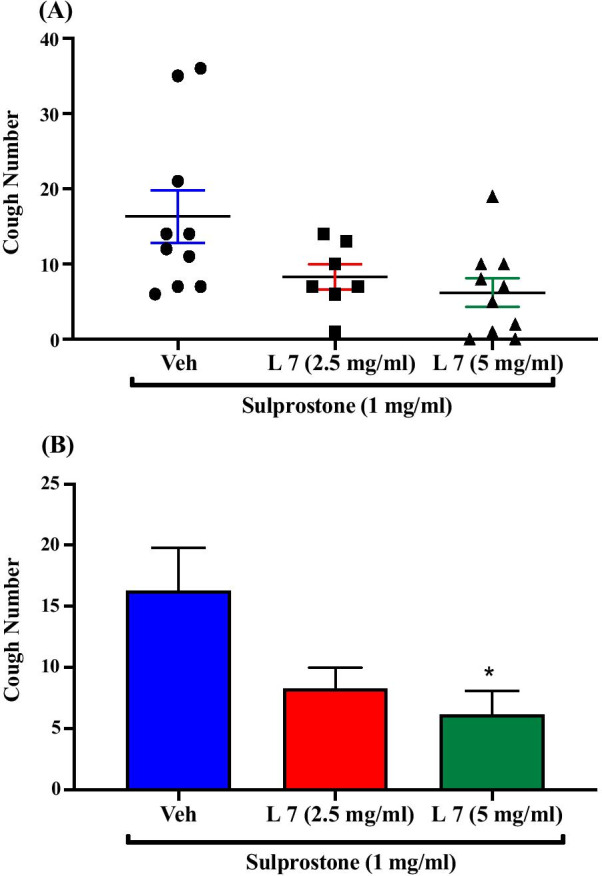
Effect of pretreatment with the EP3 antagonist, L-798,106, on sulprostone-enhanced citric acid-induced cough. Pretreatment of guinea pigs with 15 μl of L-798,106 (2.5 mg/ml; n = 7 and 5 mg/ml; n = 10, i.c.v.) resulted in a dose-dependent inhibition of the sulprostone-enhanced citric acid-induced cough response compared to vehicle (n = 10). Data are plotted as a scatter graph **A** and a bar chart **B** showing mean cough ± SEM. * represent a statistically significant difference at P ≤ 0.05 when compared to vehicle pretreated animals

Pretreatment of guinea pigs with the EP3 antagonist, L-798,106; i.c.v. (2.5 mg/ml; n = 7, 5 mg/ml; n = 10) resulted in a dose-dependent inhibition of the sulprostone-enhancement of citric acid-induced cough response compared to vehicle pretreated guinea pigs (n = 10). The mean cough ± SEM were the following: 8.29 ± 1.69 (2.5 mg/ml L-798,106), 6.20 ± 1.88 (5 mg/ml L-798,106) and 16.30 ± 3.49 (vehicle). L-798,106 at 5 mg/ml but not 2.5 mg/ml significantly reduced the sulprostone-enhancement of citric acid-induced cough response by 62% (P = 0.022). The data are presented in Fig. 5A, B.

### Effect of treatment with the EP2 agonist, butaprost, on citric acid-induced cough

**Fig. 6:**
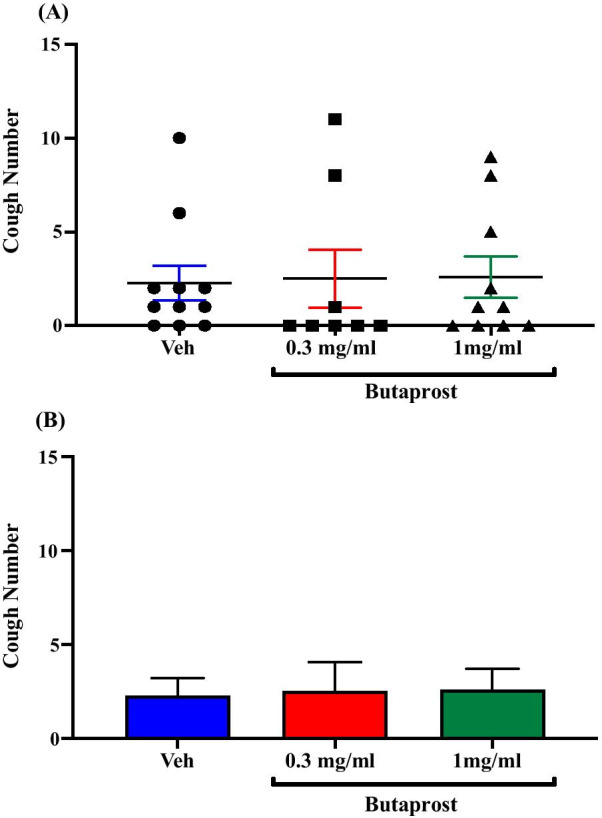
Effect of treatment with the EP2 agonist, butaprost, on citric acid-induced cough. Treatment of guinea pigs with 15 μl of butaprost (0.3 mg/ml; n = 8 and 1 mg/ml; n = 10, i.c.v.) did not affect the citric acid-induced cough response compared to vehicle (n = 11). Data are plotted as a scatter graph **A** and a bar chart **B** showing mean cough ± SEM

Treatment of guinea pigs with the selective EP2 agonist, butaprost; i.c.v. (0.3 mg/ml; n = 8 and 1 mg/ml; n = 10) did not affect the citric acid-induced cough response compared to vehicle treated guinea pigs (n = 11). The mean cough ± SEM were the following: 2.50 ± 1.56 (0.3 mg/ml butaprost), 2.60 ± 1.10 (1 mg/ml butaprost) and 2.27 ± 0.93 (vehicle). The data are presented in Fig. 6A, B.

### Effect of treatment with the EP4 agonist, L-902,688, on citric acid-induced cough

**Fig. 7:**
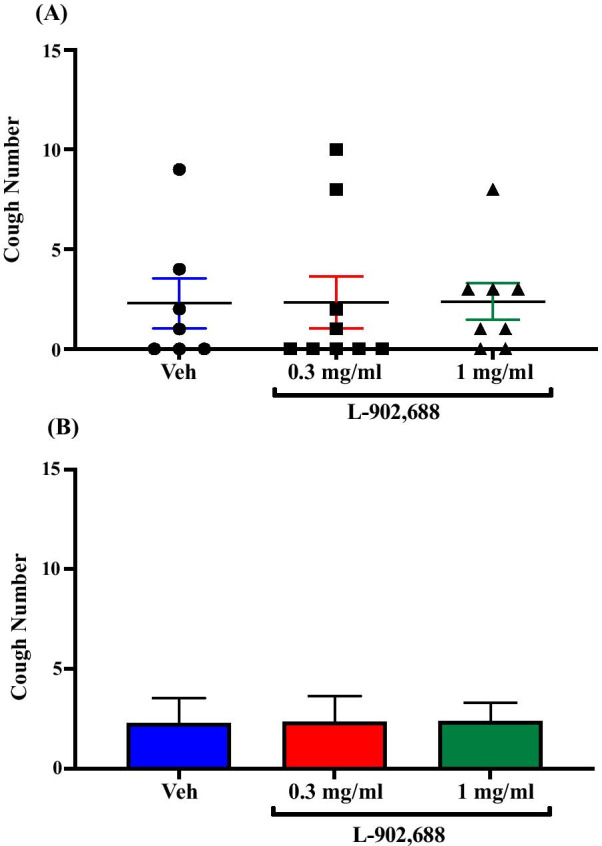
Effect of treatment with the EP4 agonist, L-902,688, on citric acid-induced cough. Treatment of guinea pigs with 15 μl of L-902,688 (0.3 mg/ml; n = 9 and 1 mg/ml; n = 8, i.c.v.) did not affect the citric acid-induced cough response compared to vehicle (n = 7). Data are plotted as a scatter graph **A** and a bar chart **B** showing mean cough ± SEM

Treatment of guinea pigs with the selective EP4 agonist, L-902,688; i.c.v. (0.3 mg/ml; n = 9 and 1 mg/ml; n = 8) did not affect the citric acid-induced cough response compared to vehicle treated guinea pigs (n = 7). The mean cough ± SEM were the following: 2.33 ± 1.29 (0.3 mg/ml L-902,688), 2.38 ± 0.92 (1 mg/ml L-902,688) and 2.29 ± 1.25 (vehicle). The data are presented in Fig. 7A, B.

### Effect of pretreatment with the TRPV1 antagonist, JNJ-17203212, on PGE_2_-enhanced citric acid-induced cough

**Fig. 8:**
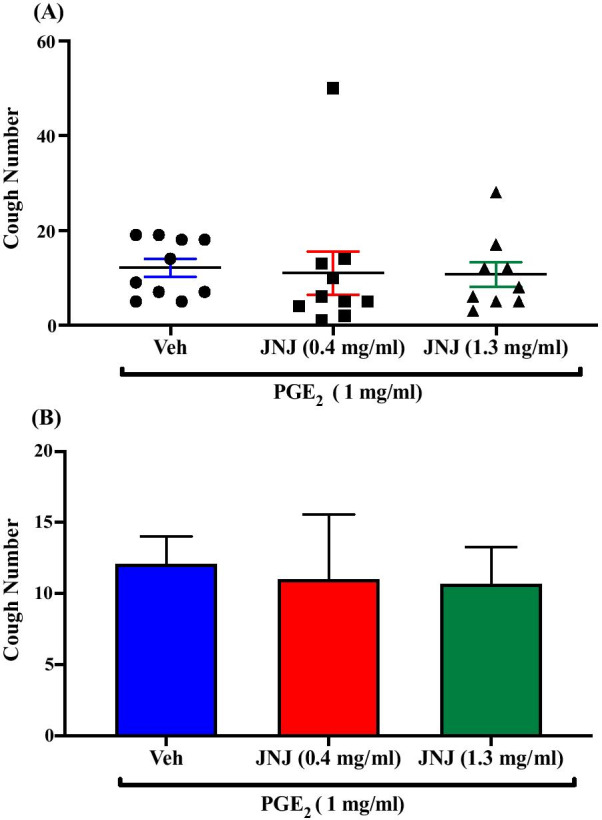
Effect of pretreatment with the TRPV1 antagonist, JNJ-17203212, on PGE_2_-enhanced citric acid-induced cough. Pretreatment of guinea pigs with 15 μl of JNJ-17203212 (0.4 mg/ml; n = 10 and 1.3 mg/ml; n = 9, i.c.v.) did not affect the PGE_2_-enhanced citric acid-induced cough response compared to vehicle (n = 10). Data are plotted as a scatter graph **A** and a bar chart **B** showing mean cough ± SEM

Pretreatment of guinea pigs with the TRPV1 channel antagonist, JNJ-17203212; i.c.v. (0.4 mg/ml; n = 10, 1.3 mg/ml; n = 9) did not affect the PGE_2_-enhancement of citric acid-induced cough response compared to vehicle pretreated guinea pigs (n = 10). The mean cough ± SEM were the following: 11.00 ± 4.55 (0.4 mg/ml JNJ-17203212), 10.67 ± 2.62 (1.3 mg/ml JNJ-17203212) and 12.10 ± 1.92 (vehicle). The data are presented in Fig. 8A, B.

### Effect of pretreatment with the TRPA1 antagonist, HC-030031, on PGE_2_-enhanced citric acid-induced cough

**Fig. 9:**
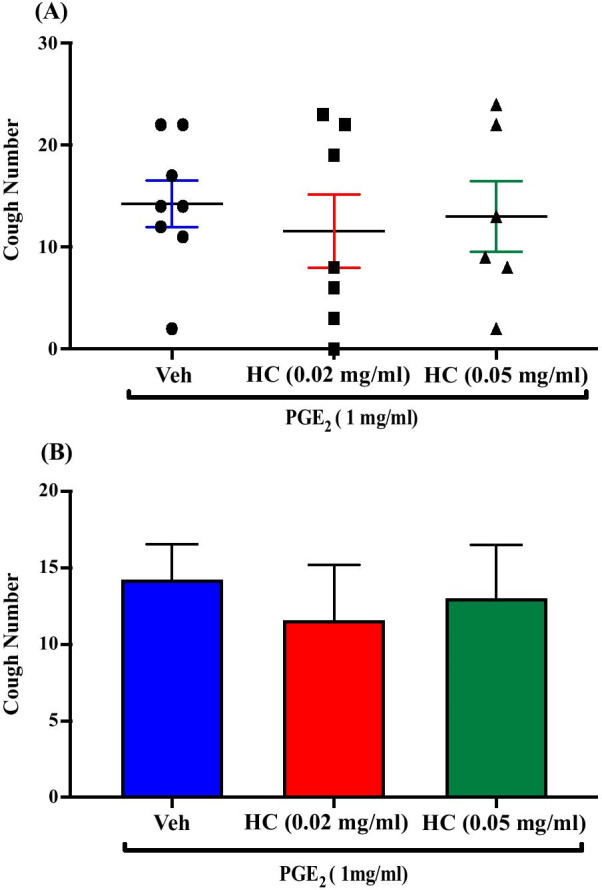
Effect of pretreatment with the TRPA1 antagonist, HC-030031, on PGE_2_-enhanced citric acid-induced cough. Pretreatment of guinea pigs with 15 μl of TRPA1 antagonist, HC-030031 (0.02 mg/ml; n = 7 and 0.05 mg/ml; n = 6, i.c.v.) did not affect the PGE_2_-enhanced citric acid-induced cough response compared to vehicle (n = 8). Data are plotted as a scatter graph **A** and a bar chart **B** showing mean cough ± SEM

Pretreatment of guinea pigs with the TRPA1 channel antagonist, HC-030031; i.c.v. (0.02 mg/ml; n = 7, 0.05 mg/ml; n = 6) did not affect the PGE_2_-enhancement of citric acid-induced cough response compared to vehicle pretreated guinea pigs (n = 8). The mean cough ± SEM were the following: 11.57 ± 3.60 (0.02 mg/ml HC-030031), 13.00 ± 3.48 (0.05 mg/ml HC-030031) and 14.25 ± 2.29 (vehicle). The data are presented in Fig. 9A, B.

### Effect of pretreatment with combined doses of JNJ-17203212 and HC-030031 on PGE_2_-enhanced citric acid-induced cough

**Fig. 10:**
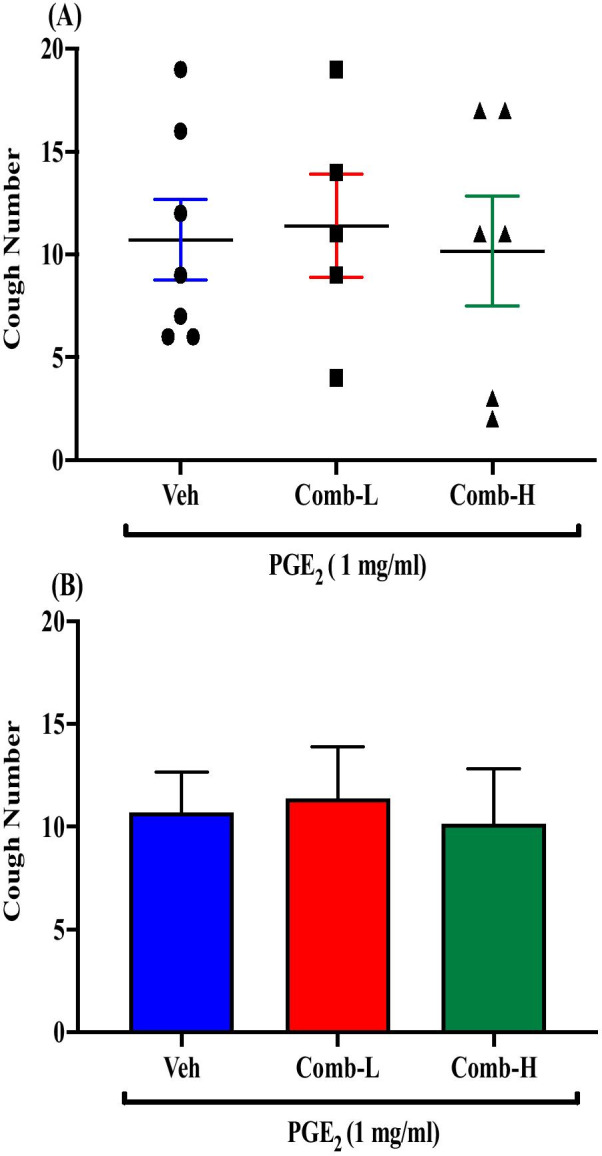
Effect of pretreatment with a combination of JNJ-17203212 and HC-030031 on PGE_2_-enhanced citric acid-induced cough. Pretreatment of guinea pigs with a 15 μl of combination of TRPV1 antagonist JNJ-17203212 and TRPA1 antagonist, HC-030031 (Comb-L: 0.4 mg/ml and 0.02 mg/ml; n = 5 and Comb-H: 1.3 mg/ml and 0.05 mg/ml; n = 6, i.c.v.) did not affect the PGE_2_-enhanced citric acid-induced cough response compared to vehicle (n = 7). Data are plotted as a scatter graph **A** and a bar chart **B** showing mean cough ± SEM

Pretreatment of guinea pigs with combined doses of JNJ-17203212 and HC-030031, i.c.v., at either low (0.4 mg/ml and 0.02 mg/ml; n = 5) or high doses (1.3 and 0.05 mg/ml; n = 6) did not affect the PGE_2_-enhancement of citric acid-induced cough response compared to vehicle pretreated guinea pigs (n = 7). The mean cough ± SEM were the following: 11.40 ± 2.50 (low-dose combination), 10.17 ± 2.66 (high-dose combination) and 10.71 ± 1.95 (vehicle). The data are presented in Fig. 10A, B.

### Effect of pretreatment with the tetrodotoxin (TTX)-sensitive channel blocker, TTX, on PGE_2_-enhanced citric acid-induced cough

**Fig. 11:**
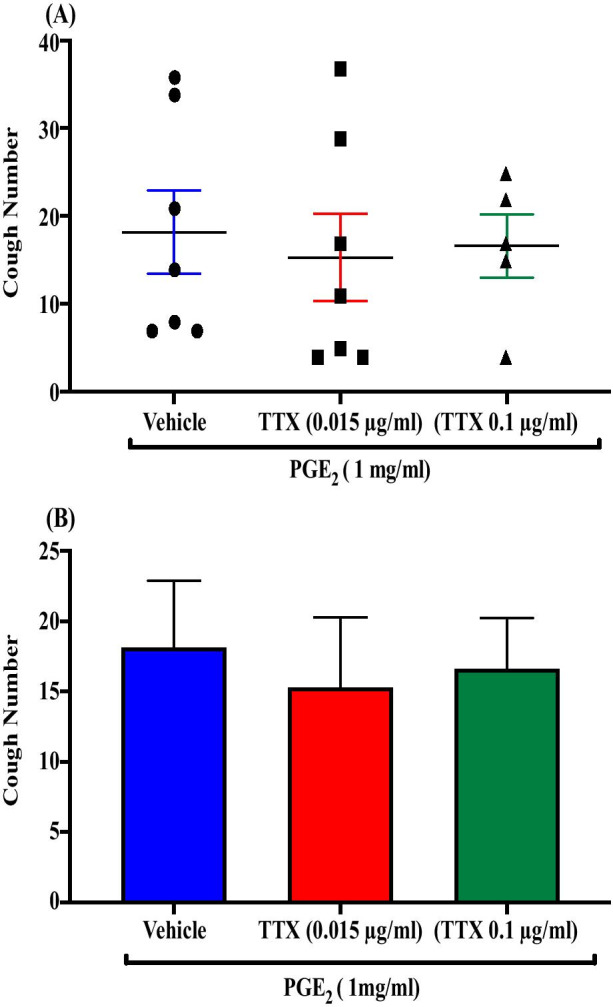
Effect of pretreatment with the tetrodotoxin (TTX)-sensitive channel blocker, TTX, on PGE_2_-enhanced citric acid-induced cough. Pretreatment of guinea pigs with 15 μl of TTX (0.015 µg/ml; n = 7 and 0.1 µg/ml; n = 5, i.c.v.) did not affect the PGE_2_-enhanced citric acid-induced cough response compared to vehicle (n = 7). Data are plotted as a scatter graph **A** and a bar chart **B** showing mean cough ± SEM

Pretreatment of guinea pigs with the tetrodotoxin, TTX; i.c.v. (0.015 µg/ml; n = 7, 0.1 µg/ml; n = 5) did not affect the PGE_2_-enhancement of citric acid-induced cough response compared to vehicle pretreated guinea pigs (n = 7). The mean cough ± SEM were the following: 15.29 ± 4.98 (0.015 µg/ml TTX), 16.60 ± 3.61 (0.1 µg/ml TTX) and 18.14 ± 4.75 (vehicle). The data are presented in Fig. 11A, B.

### Effect of pretreatment with the NaV 1.8 antagonist, A-803467, on PGE_2_-enhanced citric acid-induced cough

**Fig. 12:**
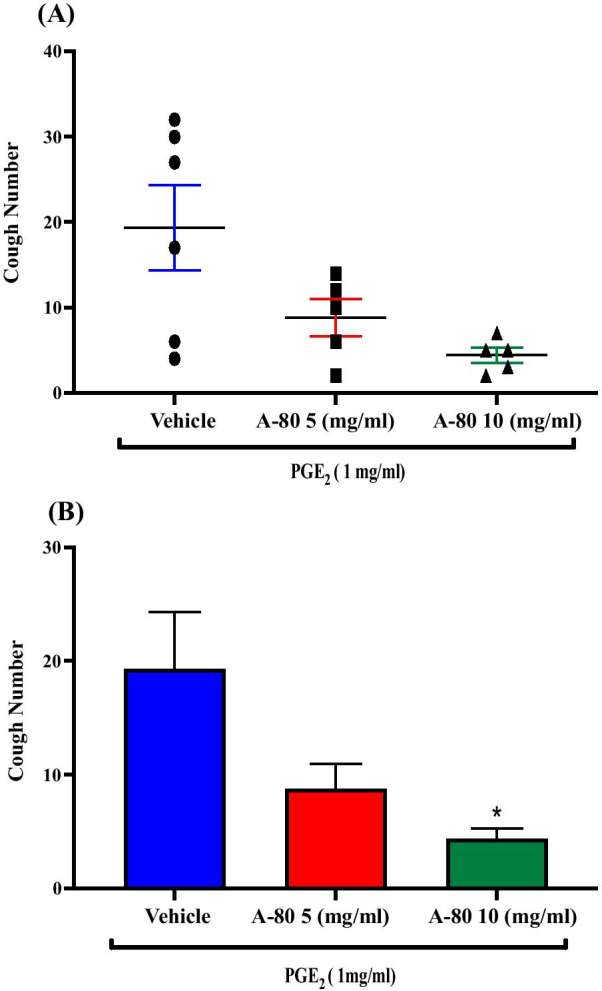
Effect of pretreatment with the NaV 1.8 antagonist, A-803467, on PGE_2_-enhanced citric acid-induced cough. Pretreatment of guinea pigs with 15 μl of A-803467 (5 mg/ml; n = 5 and 10 mg/ml; n = 5, i.c.v.) resulted in a dose-dependent inhibition of the PGE_2_-enhanced citric acid-induced cough response compared to vehicle (n = 6). Data are plotted as a scatter graph **A** and a bar chart **B** showing mean cough ± SEM. * represent a statistically significant difference at P ≤ 0.05 when compared to vehicle pretreated animals

Pretreatment of guinea pigs with the NaV1.8 channel antagonist, A-803467; i.c.v. (5 mg/ml; n = 5, 10 mg/ml; n = 5) resulted in a dose-dependent inhibition of the PGE_2_-enhancement of citric acid-induced cough response compared to vehicle pretreated guinea pigs (n = 6). The mean cough ± SEM were the following: 8.80 ± 1.79 (5 mg/ml A-803467), 4.40 ± 1.97 (10 mg/ml A-803467) and 19.33 ± 5.00 (vehicle). A-803467 at 10 mg/ml significantly reduced the PGE_2_-enhancement of citric acid-induced cough response by 77% (P = 0.018). The data are presented in Fig. 12A, B).

### The expression of NaV 1.8 channels on the brainstem nucleus, nTS of guinea pigs

**Fig. 13:**
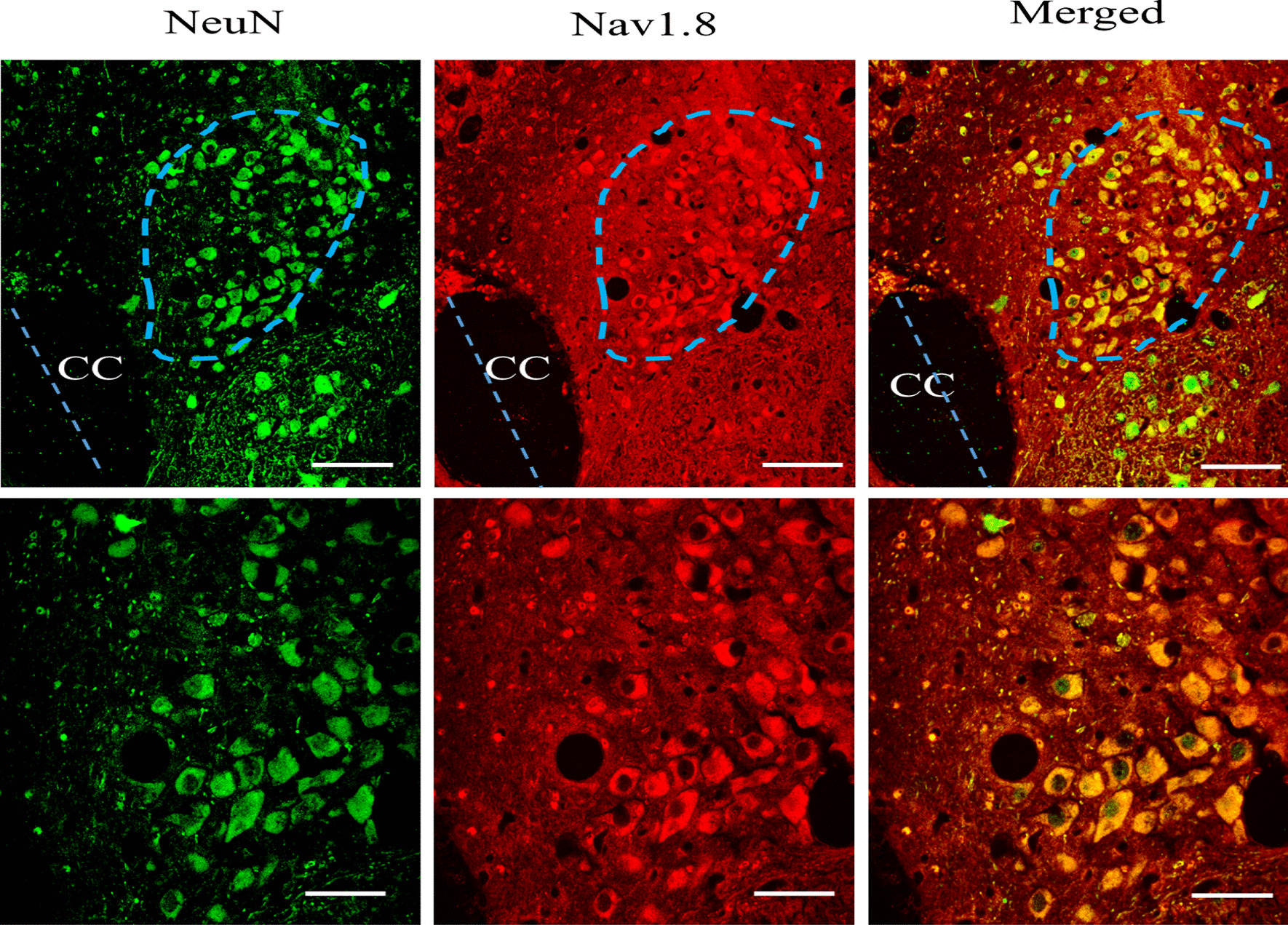
The expression of NaV 1.8 channels on the brainstem nuclei, nTS of guinea pigs. A photomicrograph of cross section of the medulla oblongata through the nucleus tractus solitarius, nTS (blue dotted line circle). A higher magnification view of the nTS is shown in the lower panel. Note all neurons of the nTS are positive for NaV 1.8. CC-Central canal. Scale bar = 15 µm in the upper panel and 30 µm in the lower panel. Blue dotted line indicates the midline

Immunohistochemistry using a polyclonal NaV 1.8 antibody confirmed the expression of NaV 1.8 channels in the nTS. NaV 1.8 channels were clearly expressed within all the neurons of the brainstem nucleus, nTS, of guinea pigs. The data are presented in Fig. 13. The nTS was identified with reference to the stereotaxic atlas of the guinea pig brainstem.

## Discussion

In this study we show, using a conscious guinea pig model of cough, that acute exposure to PGE_2_, via the central route, results in sensitization of the cough reflex which is mediated via EP3 receptor-dependent activation of NaV 1.8 channels but independently of TRPV1, TRPA1 or TTX-sensitive channel activation.

Inflammatory mediators have been reported to play a role in the sensitization of cough, at both peripheral and central levels. Elucidating the mechanism by which this is achieved will not only result in better understanding of the cough mechanisms but will also result in identification of novel targets for cough therapy.

Our data show that acute exposure to PGE_2_, via the i.c.v route, resulted in a dose-dependent enhancement of the citric acid-induced cough. The enhancement effect of PGE_2_ was observed shortly after its administration and occurs over the same time frame seen with other inflammatory mediators such as NGF and BK [12, 14]. These findings show that PGE_2_ plays a role in the central sensitization of the cough reflex, in agreement with both clinical and preclinical studies which show that PGE_2_ (peripheral) can sensitize the cough reflex/airway vagal nerves [26, 27, 35, 36]. Our findings are also in line with studies in the pain field where PGE_2_ is reported to be involved in the central mechanisms that underlie the sensitization of pain pathways leading to hyperalgesia. For example, both intrathecal (i.t.) and i.c.v. administration of PGE_2_ result in a dose-dependent enhancement of the sensitivity to pain in mice and rats [37–40].

The specific site in the brainstem where PGE_2_ acts to enhance the cough reflex has not been determined in this study. However, several studies have previously identified the nucleus tractus solitarius (nTS) as a likely site. For example, the enhanced release of several excitatory mediators of the synaptic transmission in the nTS such as glutamate and SP is proposed to result in synaptic plasticity and central sensitization of the cough reflex [14, 41–44]. Conversely, the injection of neurokinin1 (NK1) or glutamate receptor antagonists, AP-5 and CNQX, into this brain region has been reported to inhibit cough [45, 46]. Nuclei such as paratrigeminal nucleus (Pa5) may also be involved, not only as a relay point for the airway nerves, but also as a site for sensitization of the airway input signal.

All four EP receptors are reportedly expressed in the brainstem/nTS and have a high affinity for PGE_2_ [47–49] and could all potentially be involved in mediating the central tussive actions of PGE_2_. A pharmacological approach using selective agonists and also antagonists, when the selectivity of an agonist to a specific sub-type was not high, were used to rule in or out the involvement of a particular EP receptor sub-type [50].

Sulprostone, a non-selective agent with high affinity for both EP1 and EP3 receptors, was used to explore whether EP1 and/or EP3 receptors mediate the central PGE_2_ enhancement of the cough response to citric acid [47, 51]. Our data show that treatment with sulprostone, via the i.c.v. route, enhanced cough in a dose-dependent manner. Furthermore, the sulprostone enhanced cough response showed an efficacy and a time course of sensitization similar to that induced by PGE_2,_ over the same dose range_,_ suggesting that the sensitizing effects of PGE_2_ and sulprostone on cough were likely to be mediated via the same receptor subtype/s, namely EP1/EP3. These data are in line with that showing that both sulprostone and PGE_2_ induce depolarizations in the isolated guinea-pig vagus nerves, indicating that the activation of sensory nerves was also dependent on EP1/EP3 receptor subtype activation [24]. Furthermore, a role for EP1/EP3 in central sensitization is also supported by in vivo findings showing that i.t. administration of sulprostone induced hyperalgesia and allodynia, in mice and rats, with similar time course to that induced by PGE_2_ [38, 52, 53]. Altogether, these findings would suggest that PGE_2_/sulprostone mediate the enhanced citric acid-induced cough centrally via activation of EP1 and/or EP3 receptors.

To identify which one of the two receptors, or if indeed both, are involved in the sulprostone- enhanced cough, we used selective antagonists for EP1 and EP3 receptors. Our data show that pretreatment with the highly selective EP1 antagonist, ONO-8130, did not inhibit the sulprostone-enhanced citric acid-induced cough indicating that EP1 receptors were not involved in the central sensitization of the cough reflex. Higher doses of ONO-8130 could not be tested because of solubility limitations. In contrast, our data show that pretreatment with the highly selective EP3 antagonist, L-798,106 dose-dependently decreased the sulprostone-enhanced citric acid-induced cough. These findings clearly show that the effect of PGE_2_ in central sensitization of the cough reflex is mediated, at least in part, via activation of EP3 receptors. This observation is in agreement with findings from a recent study, using both in vitro and in vivo approaches, that identified EP3 as the key receptor in mediating PGE_2_-induced sensory nerve activation as well as cough induction, when administered peripherally [24]. Our results are also in line with data from pain studies which report that EP3 receptors mediate the PGE_2_-induced central sensitization of pain. For example, activation of spinal, or supra-spinal EP3 receptors had been reported to play an important role in the development of hyperalgesia and allodynia in mice and rats [38, 40, 52–54]. However, some studies have not demonstrated a role for EP3 receptors in PGE_2_- induced central sensitization of pain [55]. These differences in findings may be due to the fact that unlike other EP receptors, EP3 receptors exist in multiple splice variants isoforms [30, 56] and although these isoforms show similar ligand binding affinities, they differ in their expression between species, G-protein coupling, signal transduction properties, expression pattern and density in various tissues both in normal and disease conditions [57–59].

The highly selective EP2 agonist, butaprost (free acid, FA) and EP4 agonist, L-902,688 were used to explore whether EP2 receptors and EP4 receptors, respectively, mediate the PGE_2_ enhancement of cough response to citric acid centrally. Our data show that acute exposure to either butaprost (FA) or L-902,688, via the i.c.v route, did not affect the cough response suggesting that neither EP2 receptors nor EP4 receptors have contributed to the PGE_2_-enhanced cough centrally. These findings are in line with the results of an in vitro study which reported that neither EP2 receptors nor EP4 receptors were involved in the PGE_2_-induced depolarization of vagal sensory nerves isolated from guinea pigs and mice [24]. Our results are also in agreement with findings from several pain studies which have reported that central EP2 and EP4 receptors were not involved in central sensitization of pain. Of particular interest, are the findings of a study showing that the i.c.v administration of butaprost in rats had no effect on the nociceptive processing following mechanical and thermal stimulation [40]. Moreover, it has been shown that the spinal application of an EP4 agonist did not alter the responses of dorsal horn neurons to mechanical stimulation of the inflamed knee [59]. Some studies have nonetheless provided evidence for a role for EP2 and EP4 receptors in central sensitization of pain pathways [53, 59, 60].

We also investigated whether TRP channels, specifically TRPV1 and TRPA1, are the downstream effectors of EP3 receptor activation and central sensitization of the cough reflex. These channels have been reported to be involved in cough induced/enhanced by other inflammatory mediators as well as in EP3 receptor- mediated sensory nerve activation and peripheral cough [12, 61, 62]. Our data show that pretreatment with neither the selective and potent TRPV1 antagonist, JNJ-17203212, nor the TRPA1 antagonist, HC-030031, at doses previously shown to have significant effects [12], inhibited the PGE_2_-enhanced citric acid-induced cough. These data indicate that these channels are not involved in PGE_2_-induced central sensitization of the cough reflex. However, based on the small reduction that was noted with the individual drugs, as well as good evidence of positive interaction between TRPV1 and TRPA1 channels, we postulated that perhaps both channels may need to be blocked in order to see any significant degree of inhibition. Our data show that pretreatment with combined doses of JNJ-17203212 and HC-030031 (low and high doses) did not affect the PGE_2_-enhancement of citric acid-induced cough response thus confirming that neither TRPV1 nor TRPA1 channels are involved in PGE_2_- induced central sensitization of the cough reflex.

Given that many studies have reported a critical role for TRP channels in cough, both at peripheral and central level, this was a surprising finding [63–65]. A possible explanation is that because EP3 receptors exist in multiple isoforms coupled to different G proteins [30, 56] and have different expression patterns between peripheral and central levels in guinea pigs [66], EP3 receptors may utilize different signaling mechanisms, centrally, that do not involve activation of TRP channels. Furthermore, recent findings from clinical studies have shown that the use of different TRPV1 antagonists failed to improve cough in patients with chronic refractory cough [12, 13, 65–67], further questioning the role of TRPV1 in cough.

Finally we investigated whether voltage gated sodium channels, tetrodotoxin (TTX)-sensitive (NaV 1.1, 1.2, 1.3, 1.4, 1.6 and 1.7) and the TTX-resistant channel, specifically NaV 1.8, are coupled to EP3 receptor activation and are involved in the central sensitization of the cough reflex. The link between PGE_2_ and NaVs has been investigated because PGE_2_ was shown to potentiate both TTX-sensitive and TTX-resistant currents in different neuronal cells [67–70]. In addition, these channels, particularly NaV 1.7, 1.8 and 1.9, have been recently reported to be expressed in sensory neurons and involved in the regulation of peripheral cough [20, 22, 71, 72]. Furthermore, a large body of evidence has shown that these channels are upregulated in inflammatory conditions and in response to specific inflammatory mediators including PGE_2_ [73–77].

Our data show that pretreatment with the potent neurotoxin, TTX, via the i.c.v. route, failed to inhibit the PGE_2_-enhanced citric acid-induced cough suggesting that TTX-sensitive channels are not involved in PGE_2_-induced central sensitization of the cough reflex. This lack of effect was not dose related as TTX was shown to have clear pharmacological effects with comparable doses used in previous studies [78, 79]. Moreover, higher doses of TTX (above 0.1 µg/ml) couldn’t be tested due to observed vasomotor and respiratory side effects. In contrast to our data, peripheral cough studies have shown that pretreatment of mice with TTX significantly reduced the cough response to 0.25 M citric acid which would imply that these channels are more important peripherally [80]. Some, but not all, preclinical pain studies also support the involvement of TTX-sensitive channels in models of hyperalgesia [79]. Therefore, the precise role for TTX-sensitive channels in pain remains unclear.

A-803467 is a potent and highly selective NaV 1.8 antagonist which has been widely used to investigate the role of these channels in pain and cough studies. Our data show that pretreatment with A-803467, via the i.c.v. route, dose-dependently decreased the PGE_2_-enhanced citric acid-induced cough. This suggests that NaV 1.8 channels, (TTX-resistant sodium channels), are coupled to EP3 receptor activation and mediate the PGE_2_-induced central sensitization of the cough reflex. Our results are in agreement with a recent study reporting that both local and systemic administration of A-803467 reduced the number of coughs induced by capsaicin challenge suggesting a role of NaV 1.8 channels in peripheral cough [81]. Furthermore, findings from several in vitro and in vivo pain studies have demonstrated a critical role for NaV 1.8 channels in blocking the excitability of sensory neurons and hyperalgesia [82–84]. Interestingly, our results are also consistent with findings from pain studies suggesting a central role for NaV 1.8 channels in the sensitization of pain pathways. For example, the i.t. administration of A-803467 produced a significant effect on diabetes and cancer-induced pain in mice and rats, respectively [85, 86]. There are limited data on the expression of  NaV 1.8 channels in the CNS and specifically in the brainstem. However, , our immunohistochemistry data clearly show that NaV 1.8 channels are expressed in the brainstem, specifically in the nTS, and thus this lends further support to our pharmacological data. The expression of the NaV 1.8 channels in the nTS makes them a potential molecular target for the treatment of CHS warranting further investigation. The exact nature of the interaction and coupling between EP3 receptors and NaV 1.8 remains to be characterized. Of interest however, Kwong et al. have shown that PGE_2_ potentiates TTX-R channel function, most likely NaV 1.8 channels, in capsaicin-sensitive vagal pulmonary neurons by increasing the TTX-R conductance and increasing the voltage sensitivity; an effect that could be dependent on EP3 receptors [67, 87]. However, more work is needed to establish the nature of the interaction between EP3 and NaV 1.8 channels.

## Conclusion

In summary, our data show that PGE_2_, centrally, enhances citric acid-induced cough via EP3 receptor-dependent activation of NaV 1.8 channels but independently of TRPV1, TRPA1 and TTX-sensitive channels activation. Altogether, our findings support an important role of inflammatory mediators, specifically PGE_2_, in driving cough hypersensitivity and identify central EP3 receptors and NaV 1.8 channels as part of an important signaling pathway in enhanced cough. Therefore, targeting central EP3 receptors and/or NaV 1.8 channels may represent a novel approach for the treatment of cough hypersensitivity syndrome.

## Data Availability

The datasets generated during and/or analysed during the current study are available from the corresponding author on reasonable request.

## Abbreviations

ACE: Angiotensin Converting Enzyme
ANOVA: One-way Analysis of Variance
ACSF: Artificial Cerebrospinal Fluid
BK: Bradykinin
CO_2_: Carbon Dioxide
CHS: Cough Hypersensitivity Syndrome
DMSO: Dimethyl sulfoxide
EP: E-prostanoid Receptors
GPCR: G-protein Coupled Receptors
i.c.v.: Intracerebroventricular
i.m: Intramuscularly
i.t.: Intrathecally
NaVs: Voltage-Gated Sodium Channels
NGF: Nerve Growth Factor
NK1: Neurokinin 1
nTS: Nucleus Tractus Solitarius
Pa5: Paratrigeminal Nucleus
PBS: Phosphate Buffer Saline
PGE_2_: Prostaglandin E_2_
s.c.: Subcutaneously
SP: Substance P
TRP: Transient Resistant Potential
TTX: Tetrodotoxin
WPB: Whole Body Plethysmography

## References

[1] Morice AH, McGarvey L, Pavord I, British Thoracic Society Cough Guideline G (2006). Recommendations for the management of cough in adults. Thorax.

[2] Dicpinigaitis PV (2015). Clinical perspective - cough: an unmet need. Curr Opin Pharmacol.

[3] Chung KF (2014). Approach to chronic cough: the neuropathic basis for cough hypersensitivity syndrome. J Thorac Dis.

[4] Mazzone SB, McGovern AE, Yang SK, Woo A, Phipps S, Ando A, Leech J, Farrell MJ (2013). Sensorimotor circuitry involved in the higher brain control of coughing. Cough.

[5] Carr MJ, Undem BJ (2003). Pharmacology of vagal afferent nerve activity in guinea pig airways. Pulm Pharmacol Ther.

[6] Lieu T, Undem BJ (2011). Neuroplasticity in vagal afferent neurons involved in cough. Pulm Pharmacol Ther.

[7] Taylor-Clark TE (2015). Peripheral neural circuitry in cough. Curr Opin Pharmacol.

[8] Driessen AK, McGovern AE, Narula M, Yang SK, Keller JA, Farrell MJ, Mazzone SB (2017). Central mechanisms of airway sensation and cough hypersensitivity. Pulm Pharmacol Ther.

[9] Canning BJ (2009). Central regulation of the cough reflex: therapeutic implications. Pulm Pharmacol Ther.

[10] Ji RR (2015). Neuroimmune interactions in itch: Do chronic itch, chronic pain, and chronic cough share similar mechanisms?. Pulm Pharmacol Ther.

[11] Pacheco A (2014). Chronic cough: from a complex dysfunction of the neurological circuit to the production of persistent cough. Thorax.

[12] Al-Shamlan F, El-Hashim AZ (2019). Bradykinin sensitizes the cough reflex via a B2 receptor dependent activation of TRPV1 and TRPA1 channels through metabolites of cyclooxygenase and 12-lipoxygenase. Respir Res.

[13] El-Hashim AZ, Jaffal SM (2009). Nerve growth factor enhances cough and airway obstruction via TrkA receptor- and TRPV1-dependent mechanisms. Thorax.

[14] El-Hashim AZ, Jaffal SM, Al-Rashidi FT, Luqmani YA, Akhtar S (2013). Nerve growth factor enhances cough via a central mechanism of action. Pharmacol Res.

[15] Fox AJ, Lalloo UG, Belvisi MG, Bernareggi M, Chung KF, Barnes PJ (1996). Bradykinin–evoked sensitization of airway sensory nerves: a mechanism for ACE–inhibitor cough. Nat Med.

[16] Hewitt MM, Adams G, Mazzone SB, Mori N, Yu L, Canning BJ (2016). Pharmacology of bradykinin-evoked coughing in guinea pigs. J Pharmacol Exp Ther.

[17] Endoh T, Sato D, Wada Y, Ishihara K, Hashimoto S, Yoshinari M, Matsuzaka K, Tazaki M, Inoue T (2008). Nerve growth factor and brain-derived neurotrophic factor attenuate angiotensin-II-induced facilitation of calcium channels in acutely dissociated nucleus tractus solitarii neurons of the rat. Arch Oral Biol.

[18] Grace MS, Dubuis E, Birrell MA, Belvisi MG (2013). Pre-clinical studies in cough research: role of Transient Receptor Potential (TRP) channels. Pulm Pharmacol Ther.

[19] Materazzi S, Nassini R, Gatti R, Trevisani M, Geppetti P (2009). Cough sensors. II. Transient receptor potential membrane receptors on cough sensors. Handb Exp Pharmacol.

[20] Muroi Y, Undem BJ (2014). Targeting voltage gated sodium channels NaV1.7, Na V1.8, and Na V1.9 for treatment of pathological cough. Lung.

[21] Brozmanova M, Pavelkova N (2020). The prospect for potent sodium voltage-gated channel blockers to relieve an excessive cough. Physiol Res.

[22] Sun H, Kollarik M, Undem BJ (2017). Blocking voltage-gated sodium channels as a strategy to suppress pathological cough. Pulm Pharmacol Ther.

[23] Legler DF, Bruckner M, Uetz-von Allmen E, Krause P (2010). Prostaglandin E2 at new glance: novel insights in functional diversity offer therapeutic chances. Int J Biochem Cell Biol.

[24] Maher SA, Birrell MA, Belvisi MG (2009). Prostaglandin E2 mediates cough via the EP3 receptor: implications for future disease therapy. Am J Respir Crit Care Med.

[25] Sastre B, del Pozo V (2012). Role of PGE2 in asthma and nonasthmatic eosinophilic bronchitis. Mediators Inflamm.

[26] Choudry NB, Fuller RW, Pride NB (1989). Sensitivity of the human cough reflex: effect of inflammatory mediators prostaglandin E2, bradykinin, and histamine. Am Rev Respir Dis.

[27] Stone R, Barnes PJ, Fuller RW (1992). Contrasting effects of prostaglandins E2 and F2 alpha on sensitivity of the human cough reflex. J Appl Physiol.

[28] Sperber SJ, Hendley JO, Hayden FG, Riker DK, Sorrentino JV, Gwaltney JM (1992). Effects of naproxen on experimental rhinovirus colds. A randomized, double-blind, controlled trial. Ann Intern Med.

[29] Tenenbaum A, Grossman E, Shemesh J, Fisman EZ, Nosrati I, Motro M (2000). Intermediate but not low doses of aspirin can suppress angiotensin-converting enzyme inhibitor-induced cough. Am J Hypertens.

[30] Sugimoto Y, Narumiya S (2007). Prostaglandin E receptors. J Biol Chem.

[31] Tilley SL, Hartney JM, Erikson CJ, Jania C, Nguyen M, Stock J, McNeisch J, Valancius C, Panettieri RA, Penn RB, Koller BH (2003). Receptors and pathways mediating the effects of prostaglandin E2 on airway tone. Am J Physiol Lung Cell Mol Physiol.

[32] Mazzone SB, Mori N, Canning BJ (2005). Synergistic interactions between airway afferent nerve subtypes regulating the cough reflex in guinea-pigs. J Physiol.

[33] El-Hashim AZ, Edafiogho IO, Jaffal SM, Yousif MH, Ezeamuzie CI, Kombian SB (2011). Anti-tussive and bronchodilator mechanisms of action for the enaminone E121. Life Sci.

[34] Lewis CA, Ambrose C, Banner K, Battram C, Butler K, Giddings J, Mok J, Nasra J, Winny C, Poll C (2007). Animal models of cough: literature review and presentation of a novel cigarette smoke-enhanced cough model in the guinea-pig. Pulm Pharmacol Ther.

[35] Kwong K, Lee LY (2002). PGE(2) sensitizes cultured pulmonary vagal sensory neurons to chemical and electrical stimuli. J Appl Physiol.

[36] Lee LY, Widdicombe JG (2001). Modulation of airway sensitivity to inhaled irritants: role of inflammatory mediators. Environ Health Perspect.

[37] Minami T, Uda R, Horiguchi S, Ito S, Hyodo M, Hayaishi O (1994). Allodynia evoked by intrathecal administration of prostaglandin E2 to conscious mice. Pain.

[38] Minami T, Nishihara I, Uda R, Ito S, Hyodo M, Hayaishi O (1994). Characterization of EP-receptor subtypes involved in allodynia and hyperalgesia induced by intrathecal administration of prostaglandin E2 to mice. Br J Pharmacol.

[39] Ferreira SH, Lorenzetti BB (1996). Intrathecal administration of prostaglandin E2 causes sensitization of the primary afferent neuron via the spinal release of glutamate. Inflamm Res.

[40] Oka T, Aou S, Hori T (1994). Intracerebroventricular injection of prostaglandin E2 induces thermal hyperalgesia in rats: the possible involvement of EP3 receptors. Brain Res.

[41] Henry JL, Sessle BJ (1985). Effects of glutamate, substance P and eledoisin-related peptide on solitary tract neurones involved in respiration and respiratory reflexes. Neuroscience.

[42] Chen CY, Joad JP, Bric J, Bonham AC (2009). Central mechanisms I: plasticity of central pathways. Handb Exp Pharmacol.

[43] Bonham AC, Chen CY, Sekizawa S, Joad JP (2006). Plasticity in the nucleus tractus solitarius and its influence on lung and airway reflexes. J Appl Physiol.

[44] Mutolo D, Bongianni F, Fontana GA, Pantaleo T (2007). The role of excitatory amino acids and substance P in the mediation of the cough reflex within the nucleus tractus solitarii of the rabbit. Brain Res Bull.

[45] Canning BJ, Mori N (2010). An essential component to brainstem cough gating identified in anesthetized guinea pigs. FASEB J.

[46] Mutoh T, Bonham AC, Joad JP (2000). Substance P in the nucleus of the solitary tract augments bronchopulmonary C fiber reflex output. Am J Physiol Regul Integr Comp Physiol.

[47] Abramovitz M, Adam M, Boie Y, Carriere M, Denis D, Godbout C, Lamontagne S, Rochette C, Sawyer N, Tremblay NM, Belley M, Gallant M, Dufresne C, Gareau Y, Ruel R, Juteau H, Labelle M, Ouimet N, Metters KM (2000). The utilization of recombinant prostanoid receptors to determine the affinities and selectivities of prostaglandins and related analogs. Biochim Biophys Acta.

[48] Coleman RA, Smith WL, Narumiya S (1994). International Union of Pharmacology classification of prostanoid receptors: properties, distribution, and structure of the receptors and their subtypes. Pharmacol Rev.

[49] Laaris N, Weinreich D (2007). Prostaglandin E2 depresses solitary tract-mediated synaptic transmission in the nucleus tractus solitarius. Neuroscience.

[50] Markovic T, Jakopin Z, Dolenc MS, Mlinaric-Rascan I (2017). Structural features of subtype-selective EP receptor modulators. Drug Discov Today.

[51] Meves H (2006). The action of prostaglandins on ion channels. Curr Neuropharmacol.

[52] Nishihara I, Minami T, Uda R, Ito S, Hyodo M, Hayaishi O (1995). Effect of NMDA receptor antagonists on prostaglandin E2-induced hyperalgesia in conscious mice. Brain Res.

[53] Mebane H, Turnbach ME, Randich A (2003). Spinal EP receptors mediating prostaglandin E2-induced mechanical hyperalgesia, thermal hyperalgesia, and touch-evoked allodynia in rats. J Pain.

[54] Malmberg AB, Rafferty MF, Yaksh TL (1994). Antinociceptive effect of spinally delivered prostaglandin E receptor antagonists in the formalin test on the rat. Neurosci Lett.

[55] Kawabata A (2011). Prostaglandin E2 and pain–an update. Biol Pharm Bull.

[56] Schmid A, Thierauch KH, Schleuning WD, Dinter H (1995). Splice variants of the human EP3 receptor for prostaglandin E2. Eur J Biochem.

[57] Namba T, Sugimoto Y, Negishi M, Irie A, Ushikubi F, Kakizuka A, Ito S, Ichikawa A, Narumiya S (1993). Alternative splicing of C-terminal tail of prostaglandin E receptor subtype EP3 determines G-protein specificity. Nature.

[58] Bos CL, Richel DJ, Ritsema T, Peppelenbosch MP, Versteeg HH (2004). Prostanoids and prostanoid receptors in signal transduction. Int J Biochem Cell Biol.

[59] Bar KJ, Natura G, Telleria-Diaz A, Teschner P, Vogel R, Vasquez E, Schaible HG, Ebersberger A (2004). Changes in the effect of spinal prostaglandin E2 during inflammation: prostaglandin E (EP1-EP4) receptors in spinal nociceptive processing of input from the normal or inflamed knee joint. J Neurosci.

[60] Vuilleumier PH, Schliessbach J, Curatolo M (2018). Current evidence for central analgesic effects of NSAIDs: an overview of the literature. Minerva Anestesiol.

[61] El-Hashim AZ, Jaffal SM (2009). Nerve growth factor enhances cough and airway obstruction via TrkA receptor- and TRPV1-dependent mechanisms. Thorax.

[62] Grace M, Birrell MA, Dubuis E, Maher SA, Belvisi MG (2012). Transient receptor potential channels mediate the tussive response to prostaglandin E2 and bradykinin. Thorax.

[63] Maher SA, Grace MS, Birrell MA, Belvisi MG. Prostaglandin E2-induced sensory nerve activation is mediated by TRPA1 and TRPV1. In: D28 Itching and twitching in the airways: nerves and cough. American Thoracic Society; 2010, A5541-A5541. 10.1164/ajrccm-conference.2010.181.1_MeetingAbstracts.A5541

[64] Maher SA, Dubuis ED, Belvisi MG (2011). G-protein coupled receptors regulating cough. Curr Opin Pharmacol.

[65] Grace M, Birrell MA, Dubuis E, Maher SA, Belvisi MG (2012). Transient receptor potential channels mediate the tussive response to prostaglandin E2 and bradykinin. Thorax.

[66] Nakamura K, Kaneko T, Yamashita Y, Hasegawa H, Katoh H, Negishi M (2000). Immunohistochemical localization of prostaglandin EP3 receptor in the rat nervous system. J Comp Neurol.

[67] Kwong K, Lee LY (2005). Prostaglandin E2 potentiates a TTX-resistant sodium current in rat capsaicin-sensitive vagal pulmonary sensory neurones. J Physiol.

[68] England S, Bevan S, Docherty RJ (1996). PGE2 modulates the tetrodotoxin-resistant sodium current in neonatal rat dorsal root ganglion neurones via the cyclic AMP-protein kinase A cascade. J Physiol.

[69] Tripathi PK, Cardenas CG, Cardenas CA, Scroggs RS (2011). Up-regulation of tetrodotoxin-sensitive sodium currents by prostaglandin E(2) in type-4 rat dorsal root ganglion cells. Neuroscience.

[70] Gold MS, Levine JD, Correa AM (1998). Modulation of TTX-R INa by PKC and PKA and their role in PGE2-induced sensitization of rat sensory neurons in vitro. J Neurosci.

[71] Svajdova S, Brozmanova M (2018). Regulation of cough by voltage-gated sodium channels in airway sensory nerves. Acta Medica Martiniana.

[72] Carr MJ (2013). Regulation of cough and action potentials by voltage-gated Na channels. Pulm Pharmacol Ther.

[73] Laedermann CJ, Abriel H, Decosterd I (2015). Post-translational modifications of voltage-gated sodium channels in chronic pain syndromes. Front Pharmacol.

[74] Strickland IT, Martindale JC, Woodhams PL, Reeve AJ, Chessell IP, McQueen DS (2008). Changes in the expression of NaV1.7, NaV1.8 and NaV1.9 in a distinct population of dorsal root ganglia innervating the rat knee joint in a model of chronic inflammatory joint pain. Eur J Pain.

[75] Bennett DL, Clark AJ, Huang J, Waxman SG, Dib-Hajj SD (2019). The role of voltage-gated sodium channels in pain signaling. Physiol Rev.

[76] Zhang P, Gan YH (2017). Prostaglandin E2 upregulated trigeminal ganglionic sodium channel 1.7 involving temporomandibular joint inflammatory pain in rats. Inflammation.

[77] Lu VB, Ikeda SR, Puhl HL (2015). A 3.7 kb fragment of the mouse Scn10a gene promoter directs neural crest but not placodal lineage EGFP expression in a transgenic animal. J Neurosci.

[78] Clark WG, Coldwell BA (1973). The hypothermic effect of tetrodotoxin in the unanaesthetized cat. J Physiol.

[79] Nieto FR, Cobos EJ, Tejada MA, Sanchez-Fernandez C, Gonzalez-Cano R, Cendan CM (2012). Tetrodotoxin (TTX) as a therapeutic agent for pain. Mar Drugs.

[80] Kamei J, Nakanishi Y, Ishikawa Y, Hayashi SS, Asato M, Ohsawa M (2011). Possible involvement of tetrodotoxin-resistant sodium channels in cough reflex. Eur J Pharmacol.

[81] Brozmanova M, Svajdova S, Pavelkova N, Muroi Y, Undem BJ, Kollarik M (2019). The voltage-gated sodium channel NaV1.8 blocker A-803467 inhibits cough in the guinea pig. Respir Physiol Neurobiol.

[82] Liu C, Li Q, Su Y, Bao L (2010). Prostaglandin E2 promotes Nav1.8 trafficking via its intracellular RRR motif through the protein kinase A pathway. Traffic.

[83] Jarvis MF, Honore P, Shieh CC, Chapman M, Joshi S, Zhang XF, Kort M, Carroll W, Marron B, Atkinson R, Thomas J, Liu D, Krambis M, Liu Y, McGaraughty S, Chu K, Roeloffs R, Zhong C, Mikusa JP, Hernandez G, Gauvin D, Wade C, Zhu C, Pai M, Scanio M, Shi L, Drizin I, Gregg R, Matulenko M, Hakeem A, Gross M, Johnson M, Marsh K, Wagoner PK, Sullivan JP, Faltynek CR, Krafte DS (2007). A-803467, a potent and selective Nav1.8 sodium channel blocker, attenuates neuropathic and inflammatory pain in the rat. Proc Natl Acad Sci U S A.

[84] McGaraughty S, Chu KL, Scanio MJ, Kort ME, Faltynek CR, Jarvis MF (2008). A selective Nav1.8 sodium channel blocker, A-803467 [5-(4-chlorophenyl-N-(3,5-dimethoxyphenyl)furan-2-carboxamide], attenuates spinal neuronal activity in neuropathic rats. J Pharmacol Exp Ther.

[85] Tanaka K, Sekino S, Ikegami M, Ikeda H, Kamei J (2015). Antihyperalgesic effects of ProTx-II, a Nav1.7 antagonist, and A803467, a Nav1.8 antagonist, in diabetic mice. J Exp Pharmacol.

[86] Liu XD, Yang JJ, Fang D, Cai J, Wan Y, Xing GG (2014). Functional upregulation of nav1.8 sodium channels on the membrane of dorsal root Ganglia neurons contributes to the development of cancer-induced bone pain. PLoS ONE.

[87] Mazzone SB, Undem BJ (2009). Cough sensors. V. Pharmacological modulation of cough sensors. Handb Exp Pharmacol.

